# Book Review: Language Teacher Educator Identity

**DOI:** 10.3389/fpsyg.2021.705320

**Published:** 2021-08-23

**Authors:** Zongqiang Li

**Affiliations:** ^1^School of Education and Professional Studies, Griffith University, Brisbane, QLD, Australia; ^2^Department of Foreign Languages, Fuyang Normal University, Fuyang, China

**Keywords:** teacher identity, language teacher educator, language teacher education, pedagogy, teacher training, professional development

Gary Barkhuizen (Cambridge; New York, NY: Cambridge University Press), 2021, 92 pages, ISBNs: 9781108812665 (PB), 9781108874083 (OC)

The topic of language teacher identity has gained wide attention in language teacher education research studies from various disciplinary and theoretical perspectives. Yet, in comparison with the massive literature of previous studies on the identity of a language teacher, there are few studies on the identity of language teacher educators, who are language teachers themselves and who play a key role in language teacher education. *Language Teacher Educator Identity* by Gary Barkhuizen marks important progress on this topic. This book provides a comprehensive introduction to research studies on the identity of second language teacher educators. It aims to reveal how teacher educators construct their identities in different working environments, introduce the similarities and differences between language teacher educators in various institutional environments, and consider how teachers reflect on their teaching experiences and related teaching and research issues.

The book consists of five sections. Section one discusses who language teacher educators are by outlining the various types and definitions of teacher educators. Section two explores the construction of teacher educator identities through a study of seven language teacher educators in a doctoral programme in Colombia, presenting brief excerpts from narrative interviews and the reflections of educators on identity themes. Section three provides an overview of the steps to take to become a language teacher educator, examines the role of language teacher educators, and analyses the ways in which the activities of teacher educator intersect with their identities. The section employs eight propositions to explore teacher identity construction from the perspective of teacher educator. The fourth section explains why language teacher educators continue professional development and how their investment in further education and the negotiation of their identities intersect, focusing on the main identity-related categories of the seven teachers in the Colombian study mentioned in section two. Section five offers 40 questions to prompt further research studies on the identity of language teacher educator. The questions cover role definition, pedagogy, research, institutional service, community service, and leadership.

This book makes some contributions to the research on the identities of language teacher and teacher educator in the following ways. Above all, it includes a social justice perspective, which has seldom been mentioned in the previous studies. Regarding social justice in language education, Hawkins (2011) argues that a social justice approach changes the cognition of language education, admits inequities in the educational field, and reconstructs the role of teachers in educational change. In *Language Teacher Educator Identity*, social justice orientation is revealed to be an identity theme in the self-reflections of language teacher educators. The social justice identity of these educators is constructed through the display and practice of a moral and political stance in teacher education, encompassing the professional practices of teaching and research studies. The orientation in this book broadens the perspectives of language teacher researchers.

Moreover, the author examines the identity of language teacher educators on cognitive, social, emotional, ideological, and historical levels and emphasizes its relationship with the social, material, and technological world. This view corresponds exactly with new materialism, which critical applied linguists have advocated. Toohey (2017) argues that the research studies on the identity of language teacher from the new materialist perspective apply not only to teachers but also to the material and symbolic arrangements that assign certain identities. Language learning is a relational process that merges the material and the immaterial. In this book, language teacher educator identities dynamically interact with language teachers and material objects in classrooms, institutions, and online. Teacher educators or things are not to be viewed as isolated in an entangled world. The multiple, dynamic, and emergent identities of language teacher educators based on poststructuralist theories in this book lead readers to acknowledge the complexity of the language education landscape.

This book also demonstrates how to balance the different themes or aspects of the identities of language teacher educators. According to Norton and Early (2011), there has been much focus on the identity of learners, teachers, and teacher educators, but few research studies have been carried out on the identity of researchers. The language teacher educators in the book of Barkhuizen, which is premised on poststructuralism, have overlapping and dynamic identities including the identity of the researcher. The identities of the Columbian teacher educators are explored from the perspective of pedagogy, research study, and administrative practice. This book contributes to language teacher educator identity by classifying the service domain into two themes, creating institutional service work and community service work, such as work involving professional associations and governmental organizations. These two new identity dimensions are very useful since the “regional, political, economic, institutional,” evolving, and dynamic contexts of this book and language education “are infinitely varied globally” (p. 2). Therefore, language teacher educators are able to perform their professional work across different professional communities. The book introduces four interrelated domains of the identity work of language teacher educators, including language teacher education pedagogy, language teacher education research study/scholarship, institutional service, and leadership. These four areas basically cover the work of language teacher educators in different institutions and offer new fields for readers to explore.

This book is concise but full of essential content. Its readers, especially students and early career researchers, can quickly grasp what identity means in teacher educator discourse. However, it is a pity that this book fails to discuss the academic ethical issues involved in the study of the identity of the teacher educators due to its limited pages. Relevant content on this topic should be included in future editions. Generally speaking, the book is highly suitable for scholars who are interested in a wide range of fields, such as pedagogy and social psychology, especially doctoral students and teaching and research personnel.

## Author Contributions

The author confirms being the sole contributor of this work and has approved it for publication.

## Conflict of Interest

The author declares that the research was conducted in the absence of any commercial or financial relationships that could be construed as a potential conflict of interest.

## Publisher's Note

All claims expressed in this article are solely those of the authors and do not necessarily represent those of their affiliated organizations, or those of the publisher, the editors and the reviewers. Any product that may be evaluated in this article, or claim that may be made by its manufacturer, is not guaranteed or endorsed by the publisher.
